# Effects of Different Drought Timing on the Reduction and Control of Cadmium Uptake in Rice

**DOI:** 10.3390/toxics14040329

**Published:** 2026-04-15

**Authors:** Liqing Fu, Qiying Huang, Jiujin Lu, Jianmiao Gao, Yanfei Sheng, Nan Ye, Zhongcheng Lu, Jiawei Ma, Dan Liu, Yulei Wang

**Affiliations:** 1 Jinhua Agricultural Technology Extension and Seed Management Center, Jinhua 321000, China; fu_liqing@hotmail.com (L.F.); 15088629896@163.com (Q.H.); 18055152325@163.com (J.L.); 15925903164@163.com (Y.S.); lzc19891208@aliyun.com (Z.L.); 2 Key Laboratory of Soil Remediation and Quality Improvement in Zhejiang Province, Zhejiang A & F University, Hangzhou 311300, China; jiawma@zafu.edu.cn; 3 Zhejiang Provincial General Station for Quality of Cultivated Land and Fertilizer Management, Hangzhou 310020, China

**Keywords:** rice, drought, cadmium, uptake and translocation, DTPA-extractable, bioavailability

## Abstract

Rice is a globally important food crop, and its production is often affected by extreme climates such as drought and high temperatures. This study investigated how drought applied at different growth stages affects cadmium (Cd) uptake and accumulation in rice, as well as the underlying mechanisms. The results showed that drought treatments generally increased soil organic matter and alkali-hydrolyzed nitrogen content but decreased pH and available phosphorus content. The available Cd content in soil under the grain-filling stage drought treatment was lower than that under other treatments. Speciation analysis showed that under grain-filling stage drought, exchangeable Cd decreased by 3.04%, and residual Cd increased by 2.67%. Furthermore, drought treatments significantly enhanced soil urease and sucrase activities. Rice plant height and yield were significantly affected by the timing of drought, with the grain-filling stage drought treatment yielding the highest, while full growth stage and tillering stage drought treatments resulted in significantly lower yields. Cd content in various organs followed the order: root > stem > leaf > brown rice, with the brown rice Cd content being the lowest under grain-filling stage drought. In conclusion, drought treatment during the grain-filling stage had the least effect on Cd content in various rice tissues while maintaining a relatively high yield, providing a theoretical basis for water management in Cd-contaminated paddy fields.

## 1. Introduction

Drought is a major and serious disaster faced by agricultural production. With global warming, the intensity and frequency of drought events are gradually increasing [1]. However, rice is a water-sensitive crop, and its response to drought stress varies significantly across different growth stages, ultimately reflected in growth and development, yield components, and quality formation [2]. Cadmium (Cd) is a toxic heavy metal that poses significant risks to human health through food chain accumulation [3]. In paddy soils, Cd bioavailability is influenced by multiple interacting factors, including soil pH, organic matter content, soil texture, redox potential, and the presence of competing ions such as zinc (Zn) and iron (Fe) [4]. Simultaneously, under drought conditions, the activity of Cd in the soil increases, making it easier to transport from the underground to the above-ground parts, leading to a significant increase in Cd content in rice [5]. Therefore, controlling Cd content in rice grains is crucial for ensuring food safety and public health [6].

Soil water status is one of the key factors regulating soil Cd bioavailability and plant uptake, though it interacts with multiple soil properties, including pH, organic matter, texture, redox potential, nutrient levels, and coexisting ions [7,8]. Traditionally, flooding creates anaerobic reducing conditions, promoting Cd sulfide precipitation and decreasing Cd bioavailability [9]. Conversely, drainage or drought elevates soil redox potential, enhancing Cd transformation to exchangeable forms and increasing uptake by rice [10]. However, emerging evidence indicates that drought effects on Cd accumulation are not unidirectional but depend on timing, intensity, and duration [11,12]. For instance, drought at tillering or heading often increases soil available Cd and grain Cd [13], while moderate water deficit during late grain-filling may reduce Cd allocation to grains by modifying plant physiological processes and assimilate transport [14]. Such discrepancies highlight that drought timing is a key determinant of Cd uptake in rice, yet the underlying mechanisms remain incompletely understood.

Currently, research on the effects of water management on rice Cd uptake mostly focuses on comparisons between modes such as continuous flooding, intermittent irrigation, and moist irrigation, or on exploring the effects of drought at a specific growth stage (e.g., tillering, booting) [2,10]. Systematic studies comparing the dynamic effects of drought stress initiated at different time points throughout the entire growth cycle on Cd absorption, translocation, and accumulation are relatively scarce. Therefore, this experiment investigated the effects of different drought timings on Cd absorption and accumulation in rice and its underlying mechanisms, as well as its impact on soil heavy metal Cd, aiming to provide a scientific basis for the safe production of rice.

## 2. Materials and Methods

### 2.1. Test Materials

A pot experiment was conducted starting 15 July 2023, at the Pingshan Experimental Base of Zhejiang A & F University. The experiment was conducted outdoors under natural conditions to simulate real-world agricultural practices, though this approach inherently limits control over environmental variables such as air pollution and precipitation. The tested rice variety was the locally widely promoted hybrid rice cultivar ‘Yongyou 1540’, which has been extensively planted in mildly Cd-contaminated paddy fields across Zhejiang Province. The test soil was collected from the 0–20 cm plow layer of a historically Cd-contaminated farmland in Zhejiang Province (119.714967 E, 30.264400 N). After collection, the soil was air-dried naturally, stones and plant residues were removed, and it was ground and passed through a 2 mm nylon sieve, then thoroughly mixed for later use. The basic physicochemical properties of the test soil were as follows: pH 6.93, organic matter content 18.8 g·kg^−1^, available phosphorus content 10.12 mg·kg^−1^, alkali-hydrolyzed nitrogen content 143.5 mg·kg^−1^, available potassium content 32.67 mg·kg^−1^, available Cd content 0.46 mg·kg^−1^, and total Cd content 0.81 mg·kg^−1^. According to the “Soil Environmental Quality Risk Control Standard for Agricultural Land” (GB15618-2018) [15], this soil is at a moderate pollution level. The tested biochar was prepared from waste tea tree pruning materials pyrolyzed at 550 °C for 2 h.

### 2.2. Experimental Design

The experiment consisted of six treatments: T1 (conventional management): conventional water management (shallow water layer for early tillering, water depth 3 cm, drainage and sun-drying of the field at the peak tillering stage, shallow water irrigation during booting and heading, alternating wet and dry irrigation during late grain-filling until the dough stage, drainage at the yellow maturity stage until harvest); T2: drought throughout the entire growth period; T3: drought starting from the tillering stage; T4: drought starting from the heading stage; T5: drought starting from the grain-filling stage; T6: drought starting from the dough stage. Corresponding treatment groups are shown in Table 1. Each treatment had 3 biological replicates (pots), totaling 18 pots, arranged in a completely randomized block design. The pot positions were randomly rearranged every 7 days to eliminate microenvironmental differences until harvest.

For drought treatments (T2–T6), soil water potential was used as the control index. A vacuum gauge-type soil moisture tensiometer (MP-100, Shenzhen Lingyun Technology Co., Ltd., Shenzhen, China) was inserted into each treatment pot with the probe buried at a depth of 10 cm. When the soil water potential reached −30 ± 5 kPa, deionized water was quantitatively added to moisten the soil (approximately 80% of field capacity), and this cycle was repeated. The conventional management treatment (T1) was flood-irrigated according to the conventional pattern described above.

The experimental plastic pots were 17 cm high and 21 cm in diameter, each filled with 3.0 kg of sieved soil. All treatments received the same fertilizer application: basal fertilizer was slow-release compound fertilizer (N:P2O5:K2O = 23:6:16, Shandong Zhongqi Huasheng Fertilizer Industry Co., Ltd., Linyi, China), applied at 10 t·ha^−2^ (equivalent to 3.0 g per pot) and mixed evenly with the soil during potting; tillering fertilizer was urea (N 46%), applied at 2.5 t·ha^−2^ (equivalent to 0.75 g per pot) by top-dressing 10 days after transplanting. Rice seeds were soaked, germinated, and then sown, soil-nursed, and transplanted at the two-leaf-one-heart seedling stage, with 5 hills per pot and 1 plant per hill, resulting in 5 plants per pot (each replicate was a pooled sample of 5 plants).

### 2.3. Sample Collection and Determination

#### 2.3.1. Soil Sample Collection

At rice maturity, pot soil was collected, brought back to the laboratory, air-dried, ground, and then passed through 2 mm and 0.15 mm sieves for the determination of soil physicochemical properties, enzyme activities, and Cd speciation.

#### 2.3.2. Plant Sample Collection

At maturity, whole rice plants were harvested per pot. Plants were carefully removed, rinsed with tap water to remove attached soil from the roots, soaked in 20 mmol·L^−1^ EDTA-Na_2_ for 15 min, and then rinsed with deionized water for the removal of heavy metals attached to the plant surface. Plants were separated into roots, stems, flag-1 leaf (the first fully expanded leaf below the flag leaf), flag-2 leaf, and panicles. Panicles were threshed, sun-dried, and then dehulled using a small huller to obtain brown rice. All plant samples were killed at 105 °C for 30 min, then dried at 75 °C to constant weight. Dried samples were ground finely using a plant grinder, passed through a 0.15 mm sieve, and stored in a desiccator for later analysis.

#### 2.3.3. Rice Growth

Before harvest, three representative plants were selected from each pot to measure plant height. After drying the rice samples, the yield was determined and converted to yield per unit area.

#### 2.3.4. Cd Content in Different Parts of Rice

Rice plant Cd was determined by digestion with nitric-perchloric acid. During Cd determination, the national standard reference material GBW10020 (GSB-11) was used for quality control. The measured value of the standard material was 93.0–100.9% of the standard value, and the standard deviation of parallel samples was 0.59–9.39%.

#### 2.3.5. Soil Physicochemical Properties

The determination methods followed the stipulations in “Soil and Agricultural Chemistry Analysis” [16]. Soil pH was measured using a pH meter (STARTER300, OHAUS, Parsippany, NJ, USA) at a soil-to-water ratio of 1:2.5. Organic carbon was determined by the potassium dichromate oxidation volumetric method-external heating method, soil organic matter = soil organic carbon × 1.724; soil available phosphorus by the molybdenum-antimony anti-colorimetric method using a spectrophotometer (UV-1800, Shimadzu, Japan); alkali-hydrolyzed nitrogen by the alkali hydrolysis diffusion method; available potassium by the ammonium acetate extraction-flame photometry method (ICE-3500-AAS, Thermo Fisher Scientific Inc., Waltham, MA, USA).

#### 2.3.6. Soil Cd Speciation

Soil available Cd was extracted using 0.005 M DTPA + 0.01 M CaCl_2_ + 0.1 M TEA buffer (pH 7.3) after shaking for 2 h [17]. Soil Cd speciation was determined using the Tessier five-step sequential extraction procedure [18], dividing soil Cd into five fractions: exchangeable (EXC, 1.0 M MgCl_2_, pH = 7.0, agitation for 2 h), carbonate-bound (CAR, 1.0 M CH_3_COONa, plus CH_3_COOH of pH 5, agitation for 5 h), Fe/Mn oxides bound form (OX, 0.04 M NH_2_OH·HCl in 25% CH3COOH, agitation for 6 h at temperature of 96 ± 2 °C), organic matters-bound form (OM, 0.02 M HNO_3_ and 30% H_2_O_2_, agitation for 5 h at temperature of 85 ± 2 °C) and residual form (RES, digested with HNO_3_-HCl-HClO_4_). This method is a classical approach for heavy metal fractionation analysis, which clearly distinguishes between available and stable forms of cadmium. Cd concentration in each extraction solution was determined using a graphite furnace atomic absorption spectrometer (AA-7000, Shimadzu, Japan). During Cd determination, the national standard reference material GBW07431 was used for quality control. The measured value of the standard material was 93.0–100.9% of the standard value, and the standard deviation of parallel samples was 0.59–9.39%.

#### 2.3.7. Soil Enzyme Activity Assay

Soil catalase (CAT) activity was determined by the potassium permanganate titration method; acid phosphatase (ACP) activity by the disodium phenyl phosphate colorimetric method; urease (UE) activity by the sodium phenolate-sodium hypochlorite colorimetric method; sucrase (SC) activity by the 3,5-dinitrosalicylic acid colorimetric method [19].

### 2.4. Data Processing and Analysis

All data were recorded and organized using WPS. IBM SPSS 22.0 statistical software was used for analysis of variance (ANOVA). Duncan’s multiple range test was used for mean comparison at a significance level of 5%. Origin 2021 software was used for plotting. The calculation formulas for Cd enrichment factor and translocation factor in different parts of rice are as follows.

BCF_root = Cd content in rice roots/Total soil Cd content(1)

BCF_shoot = Cd content in above-ground parts/Total soil Cd content(2)

BCF_brown rice = Cd content in brown rice/Total soil Cd content(3)

TF_root-shoot = Cd content in above-ground parts of rice/Cd content in rice roots(4)

TF_shoot-brown rice = Cd content in brown rice/Cd content in above-ground parts of rice(5)

## 3. Results

### 3.1. Effects of Drought Timing on Rice Growth

As shown in Table 2, under conventional water management, the T1 (Conventional management) treatment had the highest plant height at 77.85 cm. Under drought conditions, grain-filling (T5) and dough stage (T6) drought had little effect on plant height, with the T5 treatment having the highest plant height value of 75.32 cm, an increase of 9.71–26.91% compared to T2, T3, and T4 treatments. Yield was highest for T5 (537.26 kg·667 m^−2^), significantly higher than the T1 and other treatments, with a 28.86% increase compared to T1 (*p* < 0.05).

### 3.2. Effects of Drought Timing on Cadmium Content in Different Parts of Rice

As shown in Table 3, except for the T6 treatment, the Cd content in rice roots of other treatments increased compared to T1, with an increase range of 18.75–43.36%; the treatment with the lowest stem Cd content was T3, at 0.60 mg·kg^−1^, a significant decrease of 51.22% compared to T1 (*p* < 0.05). The flag-1 leaf Cd content of T2, T3, and T6 treatments increased significantly compared to T1, with increases of 21.33%, 29.76%, and 28.05% respectively (*p* < 0.05); the treatment with the lowest flag-2 leaf Cd content was T5, at 0.58 mg·kg^−1^, a decrease of 6.45% compared to T1, with no significant differences in other treatments.

The brown rice Cd content of the grain-filling stage drought (T5) treatment, although slightly higher than that of T1, was significantly lower than all other drought treatments, and its increase was much lower than that of other early drought treatments. The brown rice Cd content of the dough stage drought (T6) treatment was also relatively low. This indicates that implementing drought during and after the grain-filling stage greatly weakens the “promoting” effect on brown rice Cd accumulation.

### 3.3. Effects of Drought Timing on Cadmium Enrichment and Transport Coefficients in Rice

In Table 4, the highest BCF_root value was for the T5 treatment at 3.96, an increase of 18.43% compared to the T1 treatment group; the BCF_shoot value for T5 was the lowest, a decrease of 18.48% compared to T1 (*p* < 0.05); the BCF_grain value for T1 was 0.39, lower than other treatments, and significantly different from T2, T3, T4, and T6 treatments (*p* < 0.05). The TF_root-shoot value for T3 was 0.68, the lowest among all treatments, a decrease of 36.45% compared to T1 (*p* < 0.05). The analysis of TF_shoot-grain showed that the TF_shoot-grain value for T1 was 0.11, significantly lower than all other treatments, with decreases of 38.89%, 50.00%, 54.17%, 38.89%, and 35.29%, respectively (*p* < 0.05).

### 3.4. Effects of Drought Timing on Soil Physicochemical Properties

As shown in Table 5, soil pH values for other treatments decreased compared to T5, with a reduction range of 0.04–0.31 units; soil organic matter content for T4–T6 treatments increased significantly compared to T1, with increases of 3.41%, 4.26%, and 5.64% respectively (*p* < 0.05); the decrease in soil alkali-hydrolyzed nitrogen content was most significant in the T2 treatment, a decrease of 30.80% compared to T1 (*p* < 0.05); except for T6, soil available phosphorus content in other treatments was significantly lower than T1, with decreases of 13.47–53.10% (*p* < 0.05). The soil available potassium content under T2 treatment was the highest at 97.33 mg·kg^−1^, significantly higher than the T1 treatment group, an increase of 44.86% (*p* < 0.05).

### 3.5. Effects of Drought Timing on Soil Available Cadmium and Cadmium Speciation

**Figure 1:**
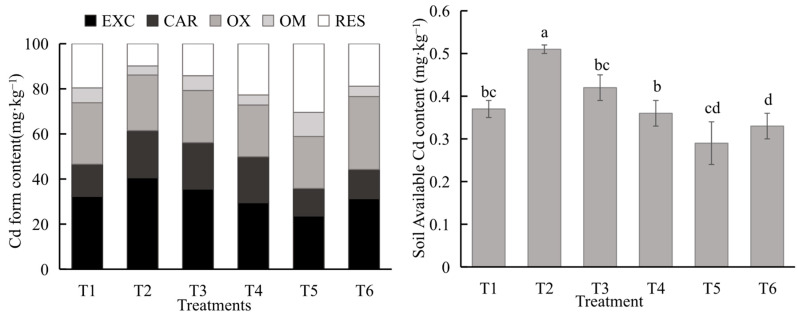
Soil available cadmium and cadmium form content. Note: EXC: exchangeable Cd; CAR: carbonate-bound Cd; OX: Fe-Mn oxide-bound Cd; OM: organic matter-bound Cd; RES: residual Cd. All error metrics are presented consistently as mean ± standard deviation, and and used Duncan’s multiple range test (*p* < 0.05), different letters within the same column indicate significant differences between treatments.

As shown in Figure 1, the proportions of soil EXC-Cd, CAR-Cd, OX-Cd, OM-Cd, and RES-Cd in the T1 treatment were 32.30%, 14.26%, 27.26%, 6.59%, and 19.58%, respectively. Compared to the conventional management group, soil EXC-Cd in the T5 treatment decreased significantly by 8.69% (*p* < 0.05); compared to the conventional management group, the CAR-Cd fraction in the T2 treatment increased the most significantly, by 6.66%; the OX-Cd fraction percentage in the T4 treatment was the lowest at 22.86%, a significant decrease of 4.31% compared to T1 (*p* < 0.05); finally, except for T2, all other treatments showed significant differences from T1 (*p* < 0.05). Among all treatments, the RES-Cd fraction percentage was highest in T5, an increase of 10.86% compared to T1 (*p* < 0.05). The soil available Cd content in the T5 treatment was the lowest, a decrease of 21.62% compared to the T1 treatment.

### 3.6. Effects of Drought Timing on Soil Enzyme Activities

The soil catalase content was highest in the T6 treatment, an increase of 2.10% compared to T1, with no significant differences in other treatments; the acid phosphatase content in T2–T4 treatments was significantly higher than in T1, with increases of 5.29%, 4.65%, and 5.33% respectively (*p* < 0.05), with the T4 treatment having the highest value of 22.52 μmol·d^−1^·g^−1^. Except for T6, soil urease content in other treatments was significantly higher than in T1, with increases of 26.60–43.71% (*p* < 0.05), with the T5 treatment having the highest value of 166.44 μg·d^−1^·g^−1^; soil sucrase content in all treatments was significantly increased compared to T1 (*p* < 0.05), with T5 having the highest sucrase content, an increase of 57.10% compared to T1 (*p* < 0.05).

### 3.7. Correlation Analysis

**Figure 2:**
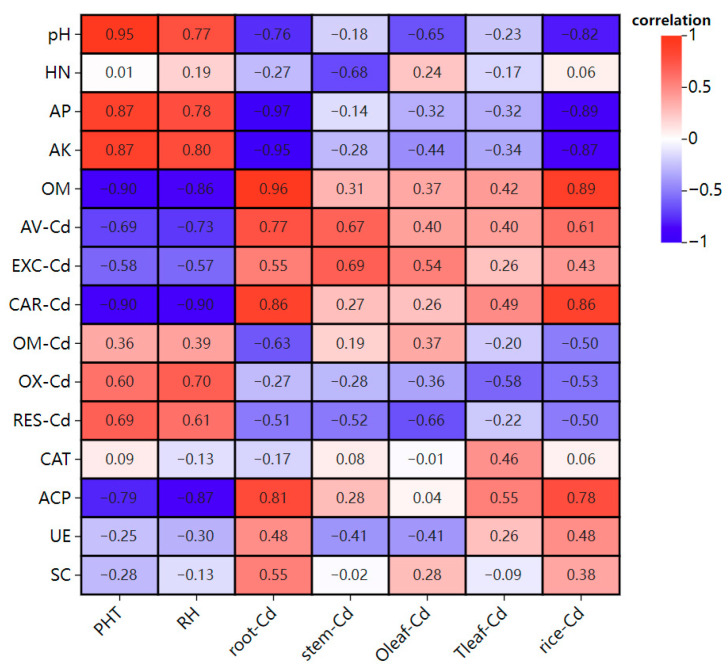
Correlation analysis. Note: HN: alkali-hydrolyzed nitrogen; AP: available phosphorus; AK: available potassium; OM: organic matter; AV-Cd: available Cd; EXC-Cd: exchangeable Cd; CAR-Cd: carbonate-bound Cd; OX-Cd: Fe-Mn oxide-bound Cd; OM-Cd: organic matter-bound Cd; RES-Cd: residual Cd; CAT: catalase; ACP: acid phosphatase; UE: urease; SC: sucrase; PHT: plant height.

In Figure 2, soil pH had a significant negative correlation with grain Cd (*p* < 0.05) and a highly significant positive correlation with rice plant height (*p* < 0.01); soil available phosphorus had a significant positive correlation with plant height (*p* < 0.05), a highly significant negative correlation with root Cd (*p* < 0.01), a significant negative correlation with grain Cd (*p* < 0.05), and a significant positive correlation with plant height (*p* < 0.05). Carbonate-bound Cd had a significant negative correlation with plant height and grain yield (*p* < 0.05), and a significant positive correlation with root Cd content and grain Cd content (*p* < 0.05). Acid phosphatase had a significant negative correlation with grain yield (*p* < 0.05). It should be noted that correlation analysis does not imply causation, and the observed relationships may be influenced by confounding factors or spurious correlations due to the limited sample size and multiple comparisons.

## 4. Discussion

### 4.1. Effects of Drought Timing on Soil Available Cadmium and Cadmium Speciation

This study confirmed that the effect of drought on soil Cd availability is time-dependent and revealed the dual mechanisms behind it. The classic view that early drought (full growth period, tillering stage) usually increases Cd availability was validated in this study (T2, T3 treatments), with the main driving factor being the decrease in soil pH, which controls chemical processes such as adsorption–desorption and precipitation-dissolution of heavy metals in soil [8,20]. However, the observed differences in soil pH among treatments were not consistently statistically significant (Table 5), suggesting that additional factors, such as changes in soil organic matter composition, microbial activity, and redox potential, may also contribute to the observed variations in Cd availability [21]. Under aerobic conditions, protons (H^+^) produced by organic matter decomposition and nitrification increase H^2+^ concentration in the soil solution. On one hand, this displaces Cd^2+^ adsorbed on colloids through ion exchange; on the other hand, it inhibits the hydrolysis and precipitation of Cd^2+^, thereby increasing its activity [22]. Simultaneously, early long-term drought may cause partial reduction and dissolution of iron-manganese oxides, releasing bound Cd, which also explains the decrease in the proportion of Fe-Mn oxide-bound Cd in the T2 treatment [23].

In this study, grain-filling stage drought (T5) achieved the “anomalous” effect of reducing soil Cd availability under drought conditions. Compared to early drought, T5 treatment maintained a relatively higher soil pH, may contribute to the transformation of Cd^2+^ to Cd(OH)_2_ precipitates, and is likely associated with the negative charge on soil colloid surfaces (e.g., organic matter, oxides), thereby fixing Cd through electrostatic and specific adsorption [24]; furthermore, under higher pH conditions, organic matter exists more in the form of humic acids, whose abundant functional groups (e.g., -COOH, -OH) can effectively fix Cd through complexation and adsorption [25]. T5 treatment significantly promoted the transformation of Cd from exchangeable to residual forms. This transformation may be related to reduced soil moisture under drought conditions, promoting the aging and crystallization of iron-manganese oxides and the co-precipitation of Cd with aluminosilicate minerals [7].

### 4.2. Effects of Drought Timing on Rice Growth and Cadmium Content in Different Parts

Agricultural production can be severely impacted by drought, leading to crop yield reduction or even failure and threatening human survival [26]. However, rice’s water demand varies across different growth stages, so the impact of drought timing on rice yield also differs [1,27]. In this study, grain yield under grain-filling stage drought treatment was significantly higher than other treatments (*p* < 0.05), with a 6.81% increase compared to the conventional management. This increase may be associated with multiple physiological adjustments, including altered source–sink relationships, enhanced remobilization of non-structural carbohydrates, and changes in phytohormone levels [12]. However, as plant hormone levels and carbohydrate dynamics were not directly measured in this study, further investigation is needed to confirm the underlying mechanisms.

Early drought stress activates Cd retranslocation from vegetative organs to grains, a stress-response mechanism that elevates grain Cd. This may be a stress “memory” or compensation mechanism. After damage to vegetative organs, plants may more efficiently reallocate resources (unfortunately, including harmful Cd) during the reproductive growth stage to ensure reproductive success [28]. Furthermore, the heading stage is a period of vigorous nitrogen metabolism and protein synthesis in rice. Cd may co-transport with nitrogen assimilates (e.g., amino acids, peptides) to grains via the phloem in the form of complexes [29]. Some studies suggest that Cd transport in the phloem is related to its chemical form and companion molecules, and the specific physiological state during grain filling may be unfavorable for the formation and transport of Cd-ligand complexes [11]. It is important to note that while Moderate grain-filling drought poses low risks to grain quality and ecosystem functions, as it is short-duration and does not disrupt key soil processes. Long-term monitoring of soil fertility and microbial communities is recommended for sustainable application. Future research should consider these aspects before recommending large-scale applications [30].

### 4.3. Effects of Drought Timing on Soil Enzyme Activities

The widespread increase in soil urease and sucrase activities (Table 6) is an important concomitant phenomenon of drought treatments. This is usually seen as a positive adaptation of soil microbial activity to initial or moderate drought stress, or as the result of relative concentration of organic substrates (e.g., root exudates, dead microorganisms) [31]. High urease activity means active nitrogen mineralization processes, which may be related to the dynamics of alkali-hydrolyzed nitrogen under drought treatments [32]. High sucrase activity reflects enhanced soil carbon cycling. However, it is noteworthy that high enzyme activity (especially sucrase) shows a significant negative correlation with low available phosphorus (AP) (Figure 2), a result consistent with the findings of Zhou Furong et al. [33]. Such correlations may reflect coordinated responses of soil microbial activity to drought rather than direct regulatory relationships. These changes in soil enzyme activity indirectly reflect that drought alters the pattern of soil nutrient cycling, which may further affect plant nutritional status and tolerance to heavy metals [34].

## 5. Conclusions

Drought timing significantly regulates the bioavailability of soil Cd. Drought during the entire growth period and tillering stage increases soil available Cd content significantly by lowering soil pH and increasing the proportion of exchangeable Cd. In contrast, grain-filling stage drought can maintain relatively high soil pH and promote the transformation of Cd into residual forms, thereby reducing soil available Cd content. Rice growth and yield respond differently to drought timing. Moderate water stress during the grain-filling stage can increase yield without affecting plant height. Brown rice Cd accumulation is controlled by both soil availability and internal plant translocation. Early drought leads to significant increases in brown rice Cd through dual pathways of “high soil availability” and “activation of Cd retranslocation to grains”. Drought during the grain filling stage achieves relatively controllable brown rice Cd content through the synergistic effects of “reducing soil availability”, “inhibiting root-shoot initial transport”, and “not strongly activating shoot-grain retranslocation”. Considering the comprehensive soil–plant system response, implementing moderate drought starting at the grain-filling stage shows promise as a water management strategy to balance yield and Cd accumulation. However, these findings are based on a single rice cultivar under outdoor pot conditions; further validation across multiple cultivars, field conditions, and broader agroecological zones is necessary, as are investigations into molecular and microbial mechanisms, to assess the broader applicability and underlying drivers of the observed patterns before recommending widespread application. Therefore, while the grain-filling stage drought strategy shows promise, its application in agricultural practice should be considered preliminary until validated under more realistic and diverse conditions.

## Figures and Tables

**Table 1:** Research and treatment on the influence of drought duration on cadmium absorption and accumulation in rice.

Code	Drought Period	Management Method	Duration of Drought (Days)
T1	/	Conventional Management	/
T2	Whole Growth Period	Drought starts after transplanting	97
T3	Starts at Tillering Stage	Drought starts 7 days after transplanting	90
T4	Starts at Heading Stage	Drought starts 37 days after transplanting	60
T5	Starts at Grain-filling Stage	Drought starts 52 days after transplanting	45
T6	Starts at Dough Stage	Drought starts 72 days after transplanting	25

**Table 2:** The Influence of drought duration on rice growth.

Treatment	Plant Height (cm)	Grain Yield (kg·667 m^−2^)
T1	77.85 ± 2.65 a	417.18 ± 86.73 b
T2	56.00 ± 2.26 bc	147.58 ± 20.43 d
T3	55.14 ± 3.18 bc	298.47 ± 67.48 c
T4	59.05 ± 2.83 c	172.33 ± 66.28 d
T5	75.32 ± 2.61 a	537.26 ± 19.66 a
T6	68.01 ± 2.62 ab	392.70 ± 53.60 bc

Note: The relatively large standard deviations for certain treatments reflect natural variability within treatments. All error metrics are presented consistently as mean ± standard deviation and used Duncan’s multiple range test (*p* < 0.05); different letters within the same column indicate significant differences between treatments.

**Table 3:** The influence of drought duration on cadmium content in different parts of rice (mg·kg^−1^).

Treatment	Root Cd Content	Stem Cd Content	Flag-1 Leaf Cd Content	Flag-2 Leaf Cd Content	Grain Cd Content
T1	2.60 ± 0.36 c	1.23 ± 0.12 a	0.59 ± 0.05 b	0.62 ± 0.06 a	0.31 ± 0.02 c
T2	4.59 ± 0.29 a	1.45 ± 0.46 a	0.75 ± 0.07 a	0.72 ± 0.23 a	0.63 ± 0.01 a
T3	3.76 ± 0.54 ab	0.60 ± 0.01 b	0.84 ± 0.01 a	0.54 ± 0.04 a	0.56 ± 0.13 ab
T4	3.71 ± 0.15 ab	0.64 ± 0.10 b	0.58 ± 0.01 b	0.74 ± 0.05 a	0.62 ± 0.01 a
T5	3.20 ± 0.09 bc	0.65 ± 0.07 b	0.60 ± 0.01 b	0.58 ± 0.03 a	0.41 ± 0.01 bc
T6	2.46 ± 0.69 c	0.66 ± 0.09 b	0.82 ± 0.01 a	0.59 ± 0.02 a	0.43 ± 0.03 bc

Note: The relatively large standard deviations for certain treatments reflect natural variability within treatments. All error metrics are presented consistently as mean ± standard deviation, and and used Duncan’s multiple range test (*p* < 0.05), different letters within the same column indicate significant differences between treatments.

**Table 4:** The influence of drought duration on cadmium enrichment and transport coefficient in rice.

Treatment	Enrichment Factor (BCF)			Translocation Factor (TF)	
	Root	Shoot	Grain	Root-Shoot	Shoot-Grain
T1	3.23 ± 0.46 a	3.41 ± 0.07 b	0.39 ± 0.02 c	1.07 ± 0.13 a	0.11 ± 0.01 d
T2	3.81 ± 3.31 a	4.50 ± 0.69 a	0.80 ± 0.02 a	0.74 ± 0.14 b	0.18 ± 0.03 bc
T3	3.11 ± 2.73 a	3.16 ± 0.16 bc	0.70 ± 0.16 a	0.68 ± 0.04 b	0.22 ± 0.04 ab
T4	3.09 ± 2.68 a	3.23 ± 0.14 bc	0.77 ± 0.01 a	0.70 ± 0.01 b	0.24 ± 0.01 a
T5	3.96 ± 0.12 a	2.78 ± 0.05 c	0.51 ± 0.01 bc	0.70 ± 0.02 b	0.18 ± 0.01 bc
T6	3.06 ± 0.91 a	3.11 ± 0.17 bc	0.54 ± 0.04 b	1.06 ± 0.24 a	0.17 ± 0.01 c

Note: The relatively large standard deviations for certain treatments reflect natural variability within treatments. All error metrics are presented consistently as mean ± standard deviation, and and used Duncan’s multiple range test (*p* < 0.05), different letters within the same column indicate significant differences between treatments.

**Table 5:** The influence of drought duration on the physical and chemical properties of soil.

Treatment	pH	Organic Matter (g·kg^−1^)	Alkali-Hydrolyzed N (mg·kg^−1^)	Available P (mg·kg^−1^)	Available K (mg·kg^−1^)	Available Cd (mg·kg^−1^)
T1	6.69 ± 0.06 ab	13.05 ± 0.18 d	64.94 ± 1.20 a	10.17 ± 0.76 a	53.67 ± 3.21 d	0.37 ± 0.02 bc
T2	6.42 ± 0.16 b	13.28 ± 0.16 cd	44.94 ± 1.58 d	4.77 ± 0.66 e	97.33 ± 3.51 a	0.51 ± 0.01 a
T3	6.42 ± 0.08 b	13.34 ± 0.29 bcd	47.65 ± 0.70 d	6.32 ± 0.44 d	85.00 ± 2.65 b	0.42 ± 0.03 b
T4	6.56 ± 0.09 ab	13.51 ± 0.11 abc	52.77 ± 1.94 c	7.77 ± 0.76 c	75.50 ± 0.71 c	0.36 ± 0.03 c
T5	6.73 ± 0.11 a	13.63 ± 0.15 ab	57.31 ± 1.49 b	8.80 ± 0.73 bc	58.00 ± 3.00 d	0.29 ± 0.05 cd
T6	6.61 ± 0.23 ab	13.83 ± 0.11 a	63.51 ± 0.67 a	9.30 ± 0.64 ab	55.00 ± 1.00 d	0.33 ± 0.03 d

Note: The relatively large standard deviations for certain treatments reflect natural variability within treatments. All error metrics are presented consistently as mean ± standard deviation, and and used Duncan’s multiple range test (*p* < 0.05), different letters within the same column indicate significant differences between treatments.

**Table 6:** The influence of drought duration on soil enzyme activity.

Treatment	Catalase (μmol·d^−1^·g^−1^)	Acid Phosphatase (μmol·d^−1^·g^−1^)	Urease (μg·d^−1^·g^−1^)	Sucrase (mg·d^−1^·g^−1^)
T1	69.12 ± 3.09 a	21.32 ± 0.07 ab	93.69 ± 0.20 c	15.61 ± 0.31 d
T2	69.91 ± 0.98 a	22.51 ± 1.07 a	127.65 ± 4.62 b	35.00 ± 1.15 a
T3	68.33 ± 2.26 a	22.36 ± 0.50 a	131.10 ± 0.62 b	28.41 ± 0.08 b
T4	69.87 ± 0.69 a	22.52 ± 0.37 a	164.62 ± 3.82 a	21.61 ± 0.68 c
T5	69.44 ± 0.51 a	20.50 ± 0.44 b	166.44 ± 1.32 a	36.39 ± 3.98 a
T6	70.6 ± 0.22 a	20.30 ± 0.20 b	97.58 ± 2.04 c	25.12 ± 1.21 bc

Note: The relatively large standard deviations for certain treatments reflect natural variability within treatments. All error metrics are presented consistently as mean ± standard deviation, and and used Duncan’s multiple range test (*p* < 0.05), different letters within the same column indicate significant differences between treatments.

## Data Availability

The original contributions presented in this study are included in the article. Further inquiries can be directed to the corresponding authors.
